# Feed, Read, and Grow: An Observational Study on the Impact of Educational Videos About Pediatric Anxiety

**DOI:** 10.7759/cureus.94724

**Published:** 2025-10-16

**Authors:** Elizabeth L Acors, Brittany Renner, Sara Worrill, Harsha Bhagtani, Silvia N Jaimes Ocazionez, Edward P Magalhaes

**Affiliations:** 1 Department of Pediatrics, Edward Via College of Osteopathic Medicine, Blacksburg, USA; 2 Department of Epidemiology and Public Health, Edward Via College of Osteopathic Medicine, Blacksburg, USA; 3 Department of Pediatrics, Mass General Brigham, Jamaica Plain, USA; 4 Department of Neuropsychiatry and Behavioral Sciences, Edward Via College of Osteopathic Medicine, Blacksburg, USA

**Keywords:** anxiety, fear, mental health, panic, panic attacks, pediatrics, separation anxiety, social anxiety

## Abstract

Introduction and aim: Anxiety is a significant mental health challenge for the youth, impacting their emotional well-being and development. To address this issue, the Feed, Read, and Grow initiative - a collaboration involving pediatricians, the Roanoke Public Library system, medical students from Edward Via College of Osteopathic Medicine, and other creative arts organizations - created short videos about panic attacks, separation anxiety, and social anxiety to educate middle- and high-school-aged students. The videos define these conditions, teach symptom recognition, and provide coping strategies. The videos were developed with input from adolescents, pediatricians, and mental health professionals to educate children about anxiety. This study aimed to identify the impact of videos about anxiety and determine their effectiveness in educating children on anxiety. Methods: Focus groups were held at two different branches of the Roanoke Public Library system during after-school programs for middle school students. Participants, aged 10-16 years, were recruited via convenience sampling. Exclusion criteria included being under 10 or over 16 years of age, incomplete surveys, or being unable to remain for the duration of the approximately 30-minute session. Three 5-minute videos were presented featuring a panel discussion with corresponding animations. Participants were then asked to complete a self-developed questionnaire evaluating their understanding of anxiety, if they found the videos engaging and informative, and their likelihood of rewatching or sharing them. The data were analyzed for enjoyment and education. Results: A total of 29 surveys were collected, with participants ranging from ages 10 to 16 years. Overall, 72.4% of our surveyed population said they would rewatch and share the video with friends. Most participants answered the learning questions correctly, showing a statistically significant increase in their ability to describe a panic attack (p=0.03, 95% CI: 0.0046, 0.0892) and social anxiety (p=0.04, 95% CI: 0.0028, 0.1234). At branch number 1, pre- and post-surveys showed that 62.5% of students would rewatch the videos, and 75% would share them, with many adjusting their understanding of social anxiety, separation anxiety, and panic attacks. Branch number 2 post-surveys showed that 92.3% of participants felt the videos were informative, 84.6% would rewatch them, and 69.2% would share them.

Conclusion: Based on the feedback collected, the Feed, Read, and Grow team has gathered invaluable insights to guide the dissemination of these videos within the community. The results indicated that most participants enjoyed the videos, expressed a willingness to share them, and demonstrated increased knowledge after watching them. By sharing these resources, the initiative aimed to be a resource for pediatricians, teachers, mental health specialists, and families to educate and support youth with social anxiety, separation anxiety, and panic attacks.

## Introduction

Feed, Read, and Grow is a multidisciplinary collaborative project involving the public library system focused on improving access to health services, food, and literacy resources in the community of Roanoke, Virginia. Public libraries play a vital role in fostering learning and community engagement [1]. They provide a welcoming space where families can connect, explore, and strengthen relationships [1]. Libraries often serve as a crucial resource for underserved families and children, offering internet access and digital tools that deliver programs that encourage healthy living and promote overall well-being [1]. The Feed, Read, and Grow program builds on this foundation by enhancing access to reliable health information to improve community well-being. Through a partnership with the Roanoke Public Library, JOI Neuron, pediatricians, and mental health professionals, a series of educational videos on various forms of anxiety is now available on YouTube.

The prevalence of mental health issues among the pediatric population has been increasing for years. One study from 2018 reported that between 2003 and 2011-2012, the prevalence of diagnosed anxiety or depression in children aged six to 17 years increased from 5.4 to 8.4% based on the National Survey of Children's Health data [2]. This trend was further exacerbated by the COVID-19 pandemic, as demonstrated by a meta-analysis consisting of 29 studies and 80,879 participants under 18 years old, wherein the estimated prevalences of clinically elevated depression and anxiety symptoms were 25.2% and 20.5%, respectively, double that of pre-pandemic estimates [3]. Anxiety is of particular interest due to its commonality and potential adverse effects. When left untreated, anxiety disorders negatively impact a child's functioning, with disruptions in physical health, academic achievement, and social skills [4]. Long-term, untreated anxiety can be associated with reduced levels of educational attainment, relationship difficulties, less life satisfaction, more chronic stress, and increased mortality [5,6]. Thus, timely intervention is critical to fostering resilience and improving quality of life.

Anxiety disorders involve feelings of excessive fear or worry, often accompanied by physical symptoms, behavioral disturbances, and functional impairment. Common types include generalized anxiety disorder, panic disorder, social anxiety, separation anxiety, and specific phobias [7]. These conditions stem from genetic, environmental, and neurological factors, making them treatable through various interventions, such as cognitive-behavioral therapy (CBT), education, and medication [8]. Despite multiple effective treatment options being available, anxiety rates among youth continue to climb. The children and adolescents in this nation need the appropriate resources and education regarding the growing mental health crisis. This starts in the local community, which Feed, Read, and Grow’s mission targets.

This program aims to create accessible educational resources developed by professionals for use by community members. In collaboration with JOI Neuron and Roanoke Public Libraries, we developed six short videos addressing three types of anxiety as follows: panic attacks, separation anxiety, and social anxiety. Our goal was to tailor the video content toward their intended audience. While they are not addressed in this study, three of the videos targeted elementary-aged children, featuring original songs and stories by Carol Joy to portray the educational content more engagingly and entertainingly for children. Studies show music is a powerful strategy for coping [9], educating [10], and reducing anxiety; thus, it played a central part in all six of the 5-minute-long videos [11]. Digital storytelling was the other key tool to make the learning experience more fun and memorable. A case study of a club in Indonesia that uses digital storytelling for teaching in early childhood stated that many teachers use digital storytelling as it makes the content more “entertaining, captivating, engaging, communicative, and theatrical” [12]. To meet the differing developmental needs of our target audiences, videos were divided into two sets as follows: one tailored for younger children and the other for adolescents.

The three videos for middle to high-school-aged students, the subject of this study, also used digital means to engage the intended audience. However, rather than educating through fun songs and storytelling, these videos consisted of a professional panel discussion with animations created by middle schoolers. This more formal approach with the panel was intended to reach a more mature audience, while the animation was designed to make the video more relatable and engaging. One study showed that an animated educational video on a specific medical condition successfully improved understanding among children and adolescents, with knowledge partially retained one year later [13]. Through both forms of videos, we hope to create a fun learning experience regarding anxiety.

One of the short videos that we presented to our focus groups addressed panic attacks in the middle and high-school-aged population. Panic disorder is a specific diagnosis requiring recurrent unexplained panic attacks with at least one month of persistent worry or another panic attack or maladaptive behavior surrounding these attacks [14]. That is distinct from a panic attack alone, which can occur with or without being a part of a panic disorder. Panic attacks can occur sporadically or with a trigger and can arise in childhood, with numerous studies reporting how the onset in adolescence is a predictor of other mental health conditions [5]. The video focuses on various coping skills, especially if deep breathing does not work to alleviate a panic attack.

Another video focused on separation anxiety, which is the fear of separation from home or a close attachment figure. It is a normal part of child development. When the fear becomes excessive, persistent, and interferes with daily life, that is when it becomes separation anxiety disorder [4]. Symptoms can include distress and worry regarding separation from home or attachment figures, refusal to go to school or leave home, fear of being alone, nightmares regarding separation, and a range of physical symptoms, such as headache, nausea, and stomachache [15]. All of these symptoms can negatively impact childhood development and daily functioning. Thus, the video we created on separation anxiety focuses on recognizing and successfully alleviating symptoms when they arise.

The last video shown discussed social anxiety. It is characterized by a persistent fear of embarrassment or humiliation in social situations and intense anxiety that affects functioning for at least six months [14]. Having social anxiety may or may not cause the patient to exhibit agoraphobic tendencies, where the patient does not leave their house or place of comfort [14]. Social anxiety is one of the most prevalent mental health conditions and often goes unrecognized and undertreated in the adolescent community [16]. Therefore, recognizing and coping with this condition is the purpose of the video.

Treatment for anxiety should encompass a combination of education, behavior-based therapy, and medication if severe. Cognitive behavioral therapy (CBT) has been demonstrated as one highly effective treatment [17]. CBT includes techniques such as restructuring anxious thoughts into more logical ones and building strategies to reduce avoidance behaviors and promote desired behaviors [17]. Importantly, all treatments pursued should be a joint effort between parent and child to achieve optimum success [4]. Despite the proven effectiveness of treatments like CBT, real-world access to care, such as this, remains limited, especially for children, a gap this project seeks to address.

Unfortunately, multiple barriers exist to assess and treat mental health disorders properly; for example, limited time, lack of experience in diagnosing anxiety disorders, concerns about stigmatizing patients, and lack of easily accessible treatment resources [18]. One unique barrier to children is that they may have difficulty accurately communicating their symptoms [4]. Our videos are designed to overcome common obstacles to mental health education by providing a free, easily accessible resource on YouTube. Backed by mental health professionals, each video is under 6 minutes long. They aim to normalize anxiety by highlighting its prevalence and featuring authentic testimonials from middle schoolers with firsthand experience. Through educating children about anxiety, including its signs and symptoms, we hope that children can learn to recognize these indicators so they can more effectively communicate their experiences to caregivers, facilitating timely intervention and treatment. Not only that, but the videos include science-backed coping strategies, such as breathing exercises [19], thinking positive thoughts [20], and seeking support [21]. Our goal through this project is to help adolescents recognize the signs and symptoms of different forms of anxiety, along with effective coping strategies. We aimed to expand access to mental health resources in underserved communities, raise awareness, and generate valuable impact data to guide future efforts. The objective of these videos was to determine their effectiveness in educating children about anxiety.

## Materials and methods

Ethical approval

This project was reviewed by the Institutional Review Board of Edward Via College of Osteopathic Medicine under project number 2037421-1. The IRB determined that this project did not meet the definition of human subject research under the purview of the IRB according to federal regulations. The quality improvement study was funded by a grant from the National Library of Medicine's network.

Participant demographics

Participants were recruited via convenience sampling through the Roanoke Public Library system, targeting students in an after-school program offered by the library. The only demographics recorded in the survey were the participants’ ages. The following inclusion criteria applied for this study: age between 10 and 16 years, as this is our target audience. The following exclusion criteria applied: those younger than 10 years or older than 16 years, unwilling to complete all parts of the survey, and/or unable to stay for the full allotted time of 30 minutes. The primary purpose of these events was to show our target audience the final videos, get their honest feedback, and use that to improve the short videos created. The data were organized into a spreadsheet and reviewed to determine if participants enjoyed the videos and would share them.

Intervention

In October and November of 2024, two focus groups were held at different branches of the Roanoke Public Library system, henceforth referred to as branch number one and branch number two, during after-school programs for middle school students. Informed assent was obtained from each participant. Participants watched three short videos on panic attacks, separation anxiety, and social anxiety. Each video featured 2-3 minutes of a small panel, which included a middle school counselor, mental health professional, and medical student discussing each type of anxiety, the signs and symptoms, and treatment. They additionally contained a 1-2 minute animation and testimonial voiceover from several middle schoolers. The three videos are included below as Videos 1-3. Participants watched each video and filled out a paper questionnaire that evaluated their understanding of each type of anxiety, whether they liked the video, their likelihood of rewatching and sharing the video with friends, and any general comments to enhance the quality and generalizability of the videos.

**Video 1:** In this video, the Feed, Read, and Grow team discusses what a panic attack is, how to know what is happening to your body during a panic attack, and what to do to help get through it.

**Video 2:** In this video, the Feed, Read, and Grow team discusses what separation anxiety is, how to recognize it, and skills to help cope.

**Video 3:** In this video, the Feed, Read, and Grow team discusses what social anxiety is, how to recognize it, and skills to help cope.

Quality improvement survey

At the focus groups, either self-developed pre- and post-surveys or only post-surveys were used to evaluate the impact of the educational videos. The learning questions in the surveys were given a score out of four to determine how many questions participants recognized as correct. Statistical significance was set at p<0.05 for these learning scores. Two pediatricians and one mental health professional reviewed the survey for validity. The questions and answer choices from the survey are included in appendix 1. For the first focus group at branch number one, participants filled out a pre-survey questionnaire consisting of three questions to assess their baseline understanding of anxiety before watching the video. The first question was a short answer assessing the participant’s understanding of a specific type of anxiety. The second and third questions were multiple choice, “select all that apply,” and asked about potential signs, symptoms, and coping mechanisms. Following the showing of the short video, participants completed a post-survey questionnaire consisting of the same questions as the pre-survey to analyze for improvement in their responses. We repeated this for all three videos. Upon completion, three additional survey questions were asked to determine their overall feelings toward the videos.

For the second focus group at branch number two, we omitted the pre-survey and adjusted the first survey question in an attempt to decrease the number of unanswered questions. The participants watched the short video and filled out the post-survey questionnaire consisting of three questions. The first question was a yes or no question asking whether the video taught them what that type of anxiety is. The second and third questions remained unchanged from the first focus group. We repeated this for all three videos, and upon completion, participants answered three additional survey questions to determine their overall feelings toward the videos.

## Results

For the first focus group at branch number one, a total of 16 survey responses were collected. The focus group at branch number two had a total of 13 survey responses collected. Out of the 29 surveys, the ages of participants ranged from 10 to 16 years old, with the most common age of 13 years (n=12). These demographics are summarized below in Table 1.

**Table 1:** Demographic information with age distribution of participants who completed the survey between the two branches (n=29). The majority of participants were 12 or 13 years old, comprising 69% of the sample. Values are presented as number (n) and percentage of total respondents.

Age (years)	n (%)
10	1 (3.45%)
11	5 (17.24%)
12	8 (27.59%)
13	12 (41.38%)
14	1 (3.45%)
15	1 (3.45%)
16	1 (3.45%)

The branch number one survey included pre- and post-surveys. For the social anxiety video, the first question was “Do you know what social anxiety is?” followed by “Did this video teach you what social anxiety is?” in the post-survey. There were several students who left this unanswered, eight in the pre-survey and 10 in the post-survey. However, some students who answered both questions have different understandings before and after the video. One student believed before the video that social anxiety is “when you feel nervous around random people,” and then their view expanded to “when a big group of people is around and you get a little nervous” after the video. Another student who had left the pre-study question unanswered then answered the post-study question with “getting nervous around other people.” The next two questions aimed to uncover what the student thought could happen to a person with social anxiety and which actions may help when a person is currently anxious. There were four options for each question, and additionally, an option for “I don’t know.” The responses are displayed in Tables 2, 3. Only four student responses of the 16 remained the same from pre- to post-survey, for a total of 25%. Of the 16 responses, six students increased their number of correct responses by one (37.5%), and three students took away a correct response after watching the video (18.75%). The remaining students maintained the same number of correct responses but adjusted the content of their responses. The coping strategy question also had four students of the 16 who maintained the same answers before and after the video (25%), while the other 12 students adjusted their responses. Two students added a correct answer (12.5%) while three students took one away (18.75%). Seven students (43.75%) maintained the same quantity of answers but changed the content of which response they selected.

**Table 2:** Answers for branch number one social anxiety video question "what are some of the things that could happen when experiencing social anxiety?." Values are presented as number (n) and percentage of total respondents.

Answers	Pre-survey, n (%)	Post-survey, n (%)
1/4 correct response	1 (6.25%)	5 (31.25%)
2/4 correct responses	3 (18.75%)	1 (6.25%)
3/4 correct responses	3 (18.75%)	5 (31.25%)
4/4 correct responses	5 (31.25%)	4 (25.00%)
"I don't know"	4 (25.00%)	1 (6.25%)
Unanswered	0 (0.00%)	0 (0.00%)

**Table 3:** Answers for branch number one social anxiety video question "which of the following actions may help if you are having social anxiety?." Values are presented as number (n) and percentage of total respondents.

Answers	Pre-survey, n (%)	Post-survey, n (%)
1/4 correct response	4 (25.00%)	4 (25.00%)
2/4 correct responses	1 (6.25%)	1 (6.25%)
3/4 correct responses	4 (25.00%)	4 (25.00%)
4/4 correct responses	6 (37.50%)	6 (37.50%)
"I don't know"	0 (0.00%)	1 (6.25%)
Unanswered	1 (6.25%)	0 (0.00%)

For the first question in the separation anxiety video, which asked students to define this condition, 10 out of 16 students left the pre-survey question unanswered, while 11 students left the post-survey question unanswered. One student who left the pre-survey question unanswered but did answer the question after the video said separation anxiety means to “sometimes be scared.” Otherwise, the students who answered the question before and after kept their answers consistent. The number of correct responses is expressed in Tables 4, 5. As far as the learning questions, the responses of five students (31.25%) remained the same from pre- to post-survey. A majority of the students who changed their answers from pre-survey to post-survey added one or more correct answers. Four students added one correct answer, while one additional student added two correct answers, for a total of 31.25% displaying an increased correctness. Two students took away a correct response, while one student took away two correct responses, for a total of 18.75%. For assessing the knowledge of coping skills, only two students maintained the same response from pre- to post-survey. Three students added one correct response, while one added two correct responses for 25% of the survey population. Two students took away one correct response, while one student took away two correct responses for 18.75% of the survey population. The remainder of the survey population (43.75%) maintained the same number of responses, but with different content.

**Table 4:** Answers for branch number one separation anxiety video question "what are some of the things that could happen when experiencing separation anxiety?." Values are presented as number (n) and percentage of total respondents.

Answers	Pre-survey, n (%)	Post-survey, n (%)
1/4 correct response	3 (18.75%)	3 (18.75%)
2/4 correct responses	0 (0.00%)	3 (18.75%)
3/4 correct responses	3 (18.75%)	3 (18.75%)
4/4 correct responses	6 (37.50%)	4 (25.00%)
"I don't know"	1 (6.25%)	1 (6.25%)
Unanswered	3 (18.75%)	2 (12.50%)

**Table 5:** Answers for branch number one separation anxiety video question "which of the following actions may help if you are having separation anxiety?." Values are presented as number (n) and percentage of total respondents.

Answers	Pre-survey, n (%)	Post-survey, n (%)
1/4 correct response	3 (18.75%)	5 (31.25%)
2/4 correct responses	0 (0.00%)	4 (25.00%)
3/4 correct responses	4 (25.00%)	4 (25.00%)
4/4 correct responses	4 (25.00%)	1 (6.25%)
"I don't know"	2 (12.50%)	0 (0.00%)
Unanswered	3 (18.75%)	2 (12.50%)

For the panic attack video responses to the first question, defining this condition, 10 pre-surveys were unanswered, while 12 post-surveys were unanswered. One student who did not answer the question in the pre-survey did note that a panic attack is “when your heart start [sic] pounding and cant [sic] breathe” after they watched the video. The number of correct responses for assessing knowledge of panic attacks and coping skills is summarized in Tables 6, 7. For the question to determine what can happen to a patient having a panic attack, five students maintained their answers between the pre- and post-surveys. Four of these five correctly selected all four out of the four correct answer choices. For the students who changed their answers from pre- to post-survey, three of the four (75%) increased their correct answers for a total of 18.75% of the 16 participants. Seven students maintained the same number of answers but changed the content. The question to assess coping skills for panic attacks only had three students who did not change their response from before the video to after. Two of these three students (66.7%) chose all four of the four correct answers. Of the students who changed their responses from pre- to post-survey, six of them increased their correct responses either by one extra correct answer or, for one student, three extra correct answers, constituting 46.15% (n=13) of participants. There were four students who decreased their correct responses, selecting fewer correct answers in the post-survey as compared to the pre-survey.

**Table 6:** Answers for branch number one panic attack video question "what are some of the things that could happen when experiencing a panic attack?." Values are presented as number (n) and percentage of total respondents.

Answers	Pre-survey, n (%)	Post-survey, n (%)
1/4 correct response	5 (31.25%)	4 (25.00%)
2/4 correct responses	1 (6.25%)	3 (18.75%)
3/4 correct responses	3 (18.75%)	3 (18.75%)
4/4 correct responses	6 (37.50%)	6 (37.50%)
"I don't know"	0 (0.00%)	0 (0.00%)
Unanswered	1 (6.25%)	0 (0.00%)

**Table 7:** Answers for branch number one panic attack video question "which of the following actions may help if you are having a panic attack?." Values are presented as number (n) and percentage of total respondents.

Answers	Pre-survey, n (%)	Post-survey, n (%)
1/4 correct response	5 (31.25%)	6 (37.50%)
2/4 correct responses	2 (12.50%)	1 (6.25%)
3/4 correct responses	3 (18.75%)	4 (25.00%)
4/4 correct responses	4 (25.00%)	4 (25.00%)
"I don't know"	0 (0.00%)	1 (6.25%)
Unanswered	2 (12.50%)	0 (0.00%)

**Figure 1:**
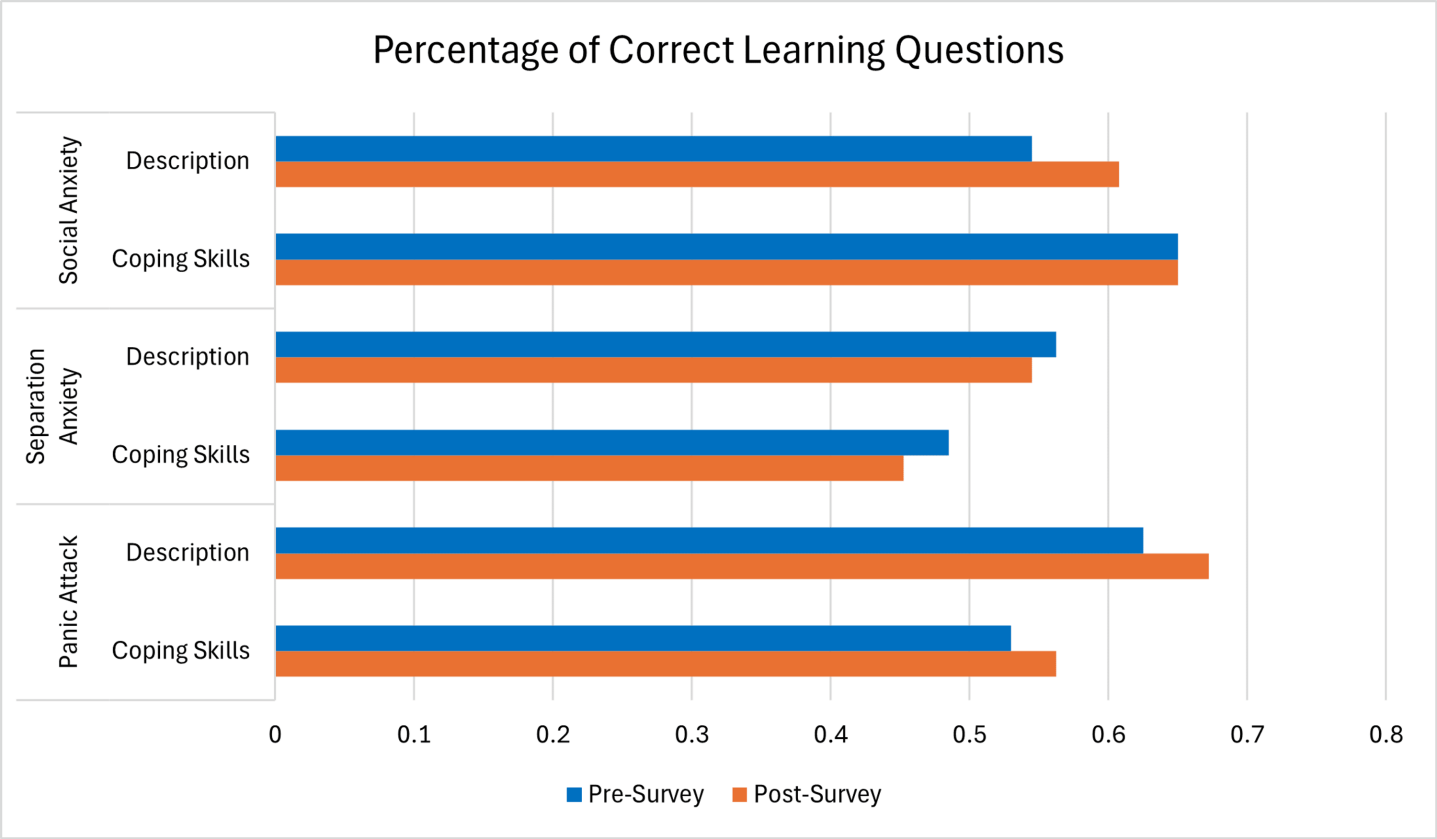
Percentage of correct responses to four learning questions for each topic (social anxiety, separation anxiety, panic attack) and question type (description, coping skills), averaged across participants (n=16). Data are represented as mean percentage of correct responses. Blue bars represent pre-survey scores and orange bars represent post-survey scores. Increases in post-survey scores suggest improved understanding following the educational videos. With a statistical significance defined as p<0.05 and the use of paired t-tests, the description of social anxiety (t=2.234, p=0.041) and panic attacks (t=2.359, p=0.030) improved significantly from pre- to post-survey.

To further evaluate changes in learning outcomes, paired t-tests were conducted to compare the pre- and post-survey scores across each video topic and question category. A statistically significant increase was found in the ability to describe a panic attack (p=0.03, 95% CI {0.0046, 0.0892}) and to describe social anxiety (p=0.04, 95% CI {0.0028, 0.1234}), suggesting improved understanding in these areas after watching the videos. No statistically significant change was observed for coping skills related to panic attacks (p=0.18), separation anxiety (p=0.22), or social anxiety (p=1.0), or for the ability to describe separation anxiety (p=0.61). The results from the three videos are summarized in Figure 1 to display the knowledge from pre- to post-survey.

Additionally, 10 of the 16 (62.5%) students said they would watch the video again, one was left unanswered, and the rest said they would not watch it again. Regarding the percentage of respondents who would share the video with others, this increased to 12 of the 16 (75%), with one unanswered and the rest as “no.”

The branch number two survey, adjusted to encourage completion, consisted only of the post-survey. Of the 13 participants, 12 (92.3%) responded “yes” when asked if each respective video taught them what social anxiety, separation anxiety, and panic attacks are. Eleven (84.6%) participants said they would watch the video again, with the remaining participants leaving the question unanswered. Nine (69.2%) participants said they would share the video with their friends, while two (15.4%) said no, and the remainder left the question unanswered. A majority of participants responded that they liked at least one video.

For the social anxiety video, when asked “what are some things that could happen when experiencing social anxiety?” eight participants got three out of the four correct answers, three participants got four out of the four correct answers, and two responded with “I don’t know.” When asked “which of the following actions may help if you are having social anxiety,” two participants got two of the four correct answers, three participants got three of the four correct answers, seven got four of the four correct answers, and one participant selected “I don’t know.” These results are summarized below in Tables 8, 9.

**Table 8:** Answers for branch number two social anxiety video question "what are some things that could happen when experiencing social anxiety?." Values are presented as number (n) and percentage of total respondents.

Answers	n (%)
1/4 correct response	0 (0.00%)
2/4 correct responses	0 (0.00%)
3/4 correct responses	8 (61.54%)
4/4 correct responses	3 (23.08%)
"I don't know"	2 (15.38%)
Unanswered	0 (0.00%)

**Table 9:** Answers for branch number two social anxiety video question "which of the following actions may help if you are having social anxiety?." Values are presented as number (n) and percentage of total respondents.

Answers	n (%)
1/4 correct response	0 (0.00%)
2/4 correct responses	2 (15.38%)
3/4 correct responses	3 (23.08%)
4/4 correct responses	7 (53.85%)
"I don't know"	1 (7.69%)
Unanswered	0 (0.00%)

For the separation anxiety videos, when asked “what are some things that could happen when experiencing separation anxiety?” two participants got two out of the four correct answers, one participant got three out of the four correct answers, eight participants got four out of the four correct answers, and two participants responded with “I don’t know.” When asked “which of the following actions may help if you are having separation anxiety,” one participant got two of the four correct answers, three participants got three of the four correct answers, six got four of the four correct answers, one participant selected “I don’t know,” and two participants left the question unanswered. These results are summarized below in Tables 10, 11.

**Table 10:** Answers for branch number two separation anxiety video question "what are some things that could happen when experiencing separation anxiety?." Values are presented as number (n) and percentage of total respondents.

Answers	n (%)
1/4 correct response	0 (0.00%)
2/4 correct responses	2 (15.38%)
3/4 correct responses	1 (7.69%)
4/4 correct responses	8 (61.54%)
"I don't know"	2 (15.38%)
Unanswered	0 (0.00%)

**Table 11:** Answers for branch number two separation anxiety video question "which of the following actions may help if you are having separation anxiety?." Values are presented as number (n) and percentage of total respondents.

Answers	n (%)
1/4 correct response	0 (0.00%)
2/4 correct responses	1 (7.69%)
3/4 correct responses	3 (23.08%)
4/4 correct responses	6 (46.15%)
"I don't know"	1 (7.69%)
Unanswered	2 (15.38%)

For the panic attack videos, when asked “what are some things that may indicate you are having a panic attack?” three participants got three out of the four correct answers, eight participants got four out of the four correct answers, and two participants left the question unanswered. When asked “which of the following actions may help if you are having a panic attack,” two participants got 0 out of the four correct answers, one participant got one out of the four correct answers, one participant got two of the four correct answers, three participants got three of the four correct answers, four got four of the four correct answers, and two participants selected “I don’t know.” These results are summarized below in Tables 12, 13.

**Table 12:** Answers for branch number two panic attack video question "what are some things that may indicate you are having a panic attack?." Values are presented as number (n) and percentage of total respondents.

Answers	n (%)
1/4 correct response	0 (0.00%)
2/4 correct responses	0 (0.00%)
3/4 correct responses	3 (23.08%)
4/4 correct responses	8 (61.54%)
"I don't know"	0 (0.00%)
Unanswered	2 (15.38%)

**Table 13:** Answers for branch number two panic attack video question "which of the following actions may help if you are having a panic attack?." Values are presented as number (n) and percentage of total respondents.

Answers	n (%)
0/4 correct responses	2 (15.38%)
1/4 correct response	1 (7.69%)
2/4 correct responses	1 (7.69%)
3/4 correct responses	3 (23.08%)
4/4 correct responses	4 (30.77%)
"I don't know"	2 (15.38%)
Unanswered	0 (0.00%)

## Discussion

The purpose of this study was to determine whether middle- and high-school-aged adolescents learned information about anxiety through the videos, enjoyed them, and would share them with others. Based on the data collected, most participants enjoyed the videos shown. The participants also indicated that they would share the videos with others.

To determine if the participants learned information about the various forms of anxiety, pre- and post-surveys at branch number one’s event helped elucidate this question, with survey responses summarized in appendix 2. Most participants either maintained the same number of correct answers or added one or more correct answers after viewing the videos, all while enjoying them. This data supports our hypothesis that the videos can provide effective education on each topic. Statistically significant improvements in the ability to describe conditions indicate that short conceptual videos can help enhance conceptual knowledge. For branch number two’s event, most of the participants accurately chose four out of the four correct answers once they watched each video, with results summarized in appendix 3. This high level of correct response rate supports the notion that adolescents were able to respond appropriately to relevant questions after watching our videos on the various subjects of panic attacks, separation anxiety, and social anxiety.

Our study findings are consistent with broader literature on digital health education in young populations. A qualitative study by Rahiem observed preschool storytelling sessions in Jakarta, Indonesia, and found that integrating digital technologies into storytelling made learning more engaging and effective for children [12]. This paper touched on the importance of incorporating digital media into education because this current generation of youth has media incorporated into every part of their lives [12]. Rahiem found that digital storytelling led to more engagement than traditional sessions and also enhanced memory retention and comprehension [12]. While our study involved an older age group, the underlying educational mechanisms likely played a similar role. Our videos, which also incorporated emotional narratives and age-appropriate visuals, may have supported the participants’ ability to describe anxiety-related conditions. This alignment with Rahiem’s findings suggests that well-designed digital media can enhance health education outcomes not only in early childhood but also in middle- and high-school populations.

Another study by Karim et al. aimed at addressing a knowledge gap in pediatric patients with chronic urticaria found that after watching a 5-minute animated educational video on the topic, participants showed a statistically significant improvement in questionnaire scores, demonstrating the video’s effectiveness in patient education [13]. Their video demonstrated a substantial increase in knowledge scores post-viewing (mean increase: 0.27, p<0.001), and they also included a retention component one year later, with retention remaining above baseline (mean: 0.83) despite limited respondent numbers [13]. Their results resonate with ours, that short, focused educational videos can drive measurable improvements in conceptual understanding in pediatric health topics. The study by Karim et al. also highlights the importance of assessing long-term retention [13]. In designing future iterations, we can build on their approach by adding reinforcement, follow-up testing, and perhaps a comparison group to isolate the effect of the video better.

A study by Schröder et al. conducted a randomized controlled trial to assess whether short YouTube videos could improve mental health literacy among adolescents and teachers [22]. Their study found that participants who watched videos on mental health demonstrated significantly greater improvements in both factual knowledge and self-reported understanding compared to those in a control group who viewed unrelated videos [22]. These findings closely parallel our own results, where short animated videos on anxiety-related topics led to measurable gains in adolescents' ability to describe social anxiety and panic attacks, as well as high levels of participant engagement. Both studies support the idea that brief, digital educational interventions can effectively enhance mental health literacy in youth, particularly when content is engaging and appropriately targeted. The encouraging results from studies like these, along with positive feedback from participants, allow the Feed, Read, and Grow team to feel confident sharing our videos with the community.

These results lay the groundwork for a future, larger research assessment, the results of which could be clinically applicable to pediatricians and mental health professionals. Osteopathic physicians may find these resources particularly useful as they reinforce the tenet that the mind, body, and spirit are interconnected [23]. Teaching pediatric patients useful coping skills early on can help them develop a stronger connection between their mental state and their physical well-being [23]. The videos created through this project are currently accessible for free online, so families can easily rewatch and share them with others. The Feed, Read, and Grow team does not have a vested interest in promoting these videos, as we do not receive any financial compensation from the viewership.

Limitations

Since we partnered with the Roanoke Public Libraries, we conducted our focus groups in this area, targeting middle schoolers from the same schools, community, and age group. As a result, our data cannot be broadly generalized to other communities with different demographics. Additionally, the limited number of responses and multiple unanswered survey questions naturally limit the extent of the conclusions that can be drawn from this evaluation. It is important to note that the videos are currently available only in English, restricting their availability for families who speak other languages or have limited familiarity with the cultural context of anxiety in the United States. Furthermore, the data collected represents only a single instance of video viewing that cannot be used to assess the program's lasting emotional impact or effectiveness in fostering long-lasting improvements in health literacy. To address these limitations, future studies could include additional focus groups in diverse locations to achieve a larger and more representative sample. Simplifying the survey questionnaire is also recommended to encourage participants to complete it fully, thereby improving the reliability and completeness of the data collected. While these videos were primarily targeted toward middle-school-aged children, they were also intended to be useful resources for caregivers, educators, and pediatricians to enhance awareness of anxiety. Although these populations were not included in this current study, future studies could focus on presenting the videos to these audiences to assess their educational impact. An additional concern is the possibility of encouraging increased screen time or causing undue anxiety about these medical conditions. By continually improving these short films, we hope to advance Feed, Read, and Grow’s mission of promoting health literacy within the Roanoke area and beyond.

## Conclusions

The primary mission of Feed, Read, and Grow is to enhance health literacy by providing easily accessible health information. The videos created through this study are available for free and aim to benefit the pediatric community through age-appropriate descriptions of mental health conditions, including social anxiety, separation anxiety, and panic attacks. While there are many other modalities of educating youth about anxiety, including group-based and problem-based interventions, this study opted to utilize a video-based intervention in order to reach our mission. Results from the two focus groups conducted showed that a majority of participants liked the videos, would share them, and displayed knowledge gains after viewing them. The Feed, Read, and Grow team has gained invaluable information to improve our future videos and ensure that the educational platform is effective in communicating information about the importance of maintaining good mental health. We hope this valuable information can reach underserved communities and be a beneficial resource utilized by pediatricians to ultimately enhance the mental health of children.

## Appendices

Appendix 1

**Table 14:** Knowledge assessment survey on panic attacks, separation anxiety, and social anxiety administered to participants. At branch number one, students completed both pre- and post-surveys; at branch number two, only the post-survey was administered. These questions aimed to assess knowledge of social anxiety, separation anxiety, and panic attacks. All multiple-response items allowed selection of one or more answers, including "I don't know" and "other." No identifying data were collected.

Questions	Answer(s)
How old are you?	
Pre-survey (only used at branch number one)	
What is social anxiety?	
What are some of the things that could happen when experiencing social anxiety? (check all that apply)	Heart could beat fast; blushing; trouble sleeping; feeling sick; I don't know; other.
Which of the following actions may help if you are having social anxiety? (check all that apply)	Use kind words to yourself; talk to someone you trust (family, doctor, teacher, counselor, etc); talk to friends; take deep breaths; I don't know; other.
What is separation anxiety?	
What are some things that could happen when experiencing separation anxiety? (check all that apply)	Feel a lot of fear; feel unhappy; feel very sad and want to cry; heart could beat fast; I don't know; other.
Which of the following actions may help if you are having separation anxiety? (check all that apply)	Remind yourself that you are strong and not alone; count things that are nearby; remember that you will see your loved one soon; talk to someone you trust (family, friends, doctor, teacher, counselor, etc); I don't know; other.
What is a panic attack?	
What are the things that may indicate you are having a panic attack? (check all that apply)	Heart could beat fast; it could be hard to breathe; feeling uncomfortable or unsettled; feeling very scared; I don't know; other.
Which of the following actions may help if you are having a panic attack? (check all that apply)	Talk to someone you trust (family, friends, doctor, teacher, counselor, etc); find something you can see, hear, smell, or touch to ground yourself; take deep breaths; think of something that will help you to calm down; I don't know; other.
Post-survey	
Did this video teach you what social anxiety is?	Yes; no
What are some of the things that could happen when experiencing social anxiety? (check all that apply)	Heart could beat fast; blushing; trouble sleeping; feeling sick; I don't know; other.
Which of the following actions may help if you are having social anxiety? (check all that apply)	Use kind words to yourself; talk to someone you trust (family, doctor, teacher, counselor, etc); talk to friends; take deep breaths; I don't know; other.
Did this video teach you what separation anxiety is?	Yes; no
What are some things that could happen when experiencing separation anxiety? (check all that apply)	Feel a lot of fear; feel unhappy; feel very sad and want to cry; heart could beat fast; I don't know; other.
Which of the following actions may help if you are having separation anxiety? (check all that apply)	Remind yourself that you are strong and not alone; count things that are nearby; remember that you will see your loved one soon; talk to someone you trust (family, friends, doctor, teacher, counselor, etc); I don't know; other.
Did this video teach you what a panic attack is?	Yes; no
What are the things that may indicate you are having a panic attack? (check all that apply)	Heart could beat fast; it could be hard to breathe; feeling uncomfortable or unsettled; feeling very scared; I don't know; other.
Which of the following actions may help if you are having a panic attack? (check all that apply)	Talk to someone you trust (family, friends, doctor, teacher, counselor, etc); find something you can see, hear, smell, or touch to ground yourself; take deep breaths; think of something that will help you to calm down; I don't know; other.
Which videos did you like? (check all that apply)	Social anxiety video; separation anxiety video; panic attack video; all of the videos; none of the videos.
Would you watch the videos again?	Yes; no
Would you share the videos with your friends?	Yes; no
Any extra comments	

Appendix 2

**Table 15:** Summary of the answers for branch number one survey determining if participants adjusted their responses between the pre- and post-survey. Values are presented as number (n) and percentage of total respondents.

Survey items	Same pre-to-post	Increased correct	Decreased correct	Same number correct, different content
“What are some of the things that could happen when experiencing social anxiety?” (16 participants)	4 (25%)	6 (37.5%)	3 (18.75)	3 (18.75)
"Which of the following actions may help if you are having social anxiety?" (16 participants)	4 (25.5%)	2 (12.5%)	3 (18.75%)	7 (43.75%)
“What are some of the things that could happen when experiencing social anxiety?” (16 participants)	5 (31.25%)	5 (31.25%)	3 (18.75%)	3 (18.75%)
"Which of the following actions may help if you are having social anxiety?" (16 participants)	2 (12.5%)	4 (25%)	3 (18.75%)	7 (43.75%)
“What are some of the things that could happen when experiencing social anxiety?” (16 participants)	5 (31.25%)	3 (18.75%)	1 (6.25%)	7 (43.75%)
"Which of the following actions may help if you are having social anxiety?" (16 participants)	3 (18.75%)	6 (37.5%)	4 (25%)	3 (18.75%)

Appendix 3

**Table 16:** Survey responses summarized from branch number two. Values are presented as number (n) and percentage of total respondents.

Answers	n, (%)
Did this video teach you what social anxiety is?	
Yes	12 (92.31%)
No	1 (7.69%)
Unanswered	0 (0.00%)
Did this video teach you what separation anxiety is?	
Yes	12 (92.31%)
No	1 (7.69%)
Unanswered	0 (0.00%)
Did this video teach you what a panic attack is?	
Yes	12 (92.31%)
No	0 (0.00%)
Unanswered	1 (7.69%)
Would you watch the videos again?	
Yes	11 (84.62%)
No	0 (0.00%)
Unanswered	2 (15.38%)
Would you share the videos with your friends?	
Yes	9 (69.23%)
No	2 (15.38%)
Unanswered	2 (15.38%)
Which videos did you like?	
All videos	5 (38.46%)
Social anxiety video	1 (7.69%)
Separation anxiety video	1 (7.69%)
Panic attack video	2 (15.38%)
Social anxiety and panic attack video	1 (7.69%)
Social anxiety video, separation anxiety video, all of the videos, none of the videos	1 (7.69%)
Unanswered	2 (15.38%)
